# Moderate coffee and tea consumption is associated with slower cognitive decline

**DOI:** 10.1177/13872877251361058

**Published:** 2025-07-21

**Authors:** Stephanie R Rainey-Smith, Kelsey R Sewell, Belinda M Brown, Hamid R Sohrabi, Ralph N Martins, Samantha L Gardener

**Affiliations:** 1 Centre for Healthy Ageing, Health Futures Institute, 5673 Murdoch University, Murdoch, Western Australia, Australia; 2 School of Psychological Science, University of Western Australia, Perth, Western Australia, Australia; 3 Centre of Excellence for Alzheimer's Disease Research and Care, School of Medical and Health Sciences, 2498 Edith Cowan University, Joondalup, Western Australia, Australia; 4 Alzheimer's Research Australia, Ralph and Patricia Sarich Neuroscience Research Institute, Nedlands, Western Australia, Australia; 5 Lifestyle Approaches Towards Cognitive Health Research Group, 5673 Murdoch University, Murdoch, Western Australia, Australia; 6 664193 AdventHealth Research Institute, Neuroscience, Orlando, FL, USA; 7 Department of Biomedical Sciences, Macquarie University, Sydney, New South Wales, Australia

**Keywords:** Alzheimer's disease, coffee, cognitive decline, tea, UK Biobank

## Abstract

**Background:**

Globally, coffee and tea are consumed extensively, potentially providing neuroprotection through anti-inflammatory and antioxidative stress effects.

**Objective:**

This study aimed to investigate associations between coffee and tea intake and cognitive function.

**Methods:**

In a longitudinal prospective cohort study, dementia-free (*n* = 8715; age range 60.0–85.2 years) older adults from the UK Biobank self-reported coffee and tea intake over the previous year; ‘never’, ‘moderate’ (1–3 cups/day), or ‘high’ (≥4 cups/day). Participants completed cognitive assessments at ≥2 timepoints (mean of 9.11 years).

**Results:**

Those ‘never’ consuming coffee and ‘moderate’ coffee consumers (*β* = 0.06, *p* = 0.005; *β* = 0.07, *p* < 0.001, respectively), as well as ‘moderate’ tea consumers and ‘high’ tea consumers (*β* = 0.06, *p* = 0.009; *β* = 0.06, *p* = 0.003, respectively) had slower fluid intelligence decline. Additionally, those ‘never’ consuming coffee and ‘moderate’ coffee consumers had a slower increase in pairs matching errors (β = −0.05, *p* = 0.022; β = 0.05, *p* = 0.013) compared to ‘high’ consumers.

**Conclusions:**

**‘**Moderate’ coffee, and ‘moderate’ and ‘high’ tea intake may be a protective factor against cognitive decline. Randomized controlled trials are required to establish causal relationships leading to evidence-based recommendations regarding benefits of coffee and tea intake.

## Introduction

Cognitive deficits associated with Alzheimer's disease represent a significant public health challenge globally, especially in aging societies.^1^ Thus, identifying modifiable factors that promote cognitive health could have wide-reaching social and economic impacts. Such modifiable factors may include coffee and tea, which are consumed extensively globally. Coffee contains biologically active substances including caffeine, chlorogenic acids, a variety of polyphenols like flavonoids, and trace amounts of vitamins and minerals,^2^ with tea containing caffeine, theanine, and flavonoids such as catechins.^3^ Coffee and tea components are thought to contribute to health benefits by providing neuroprotection through positive effects on anti-inflammatory and antioxidative stress pathways.^4^

Findings from a 2022 meta-analysis which included 22 published prospective cohort studies and 11 case-control studies, found a non-linear relationship between coffee consumption and cognitive disorder risk (including Alzheimer's disease (AD), dementia, and cognitive impairment without dementia), with a maximum of 2.5 cups/day reducing risk.^4^ In the same meta-analysis, a linear relationship between tea consumption and cognitive disorders was observed, with risk decreasing by 11% for every 1 cup/day increase. A further meta-analysis including 29 prospective studies concluded low coffee consumption reduced the risk of cognitive deficit (<2.8 cups/day) and dementia (<2.3 cups/day).^5^ Additionally, green tea consumption was a significant protective factor for cognitive health (relative risk, 0.94; 95% confidence intervals, 0.92–0.97); the linear relationship indicated that drinking one cup/day of green tea reduced the risk for cognitive deficits by 6%, whereas two cups/day reduced risk by 11%.^5^

Although substantial evidence from animal and *in vitro* studies indicates that coffee and tea exert neuroprotective effects, investigations of the possible preventative effect of coffee and tea consumption against incident cognitive impairment in humans have been divergent, likely due to confounds from dose–response effects and a lack of epidemiologic studies. Over 4.2 million person-years, 4270 dementia cases occurred among 351,436 UK Biobank participants, however, coffee consumption was not related to dementia risk, while moderate-to-high tea intake was associated with reduced incident dementia risk.^6^ A meta-analysis of eight prospective studies including 7486 dementia cases diagnosed among 328,885 individuals, during an average follow-up of 4.9–25 years, concluded the results do not support an association between coffee consumption and increased risk of overall dementia, or AD specifically.^7^ Beyond examining dementia incidence, longitudinal cohort studies with large samples are necessary to further understand the influence of coffee and tea consumption on rates of decline in distinct cognitive outcomes. Such information may aid in the development of recommendations for maintaining cognitive health with advancing age. Consequently, the aim of the current study was to investigate the relationship of self-reported habitual coffee and tea intake with rates of decline in a range of cognitive outcomes in 8715 older adults (age range 60.0–85.2 years) from the UK Biobank cohort assessed over a mean of 9.11 years (range 2.1–17.3 years), classified as dementia-free at baseline.

## Methods

### Participants

The UK Biobank is a prospective cohort of half a million middle-aged males and females recruited within the UK in 2006 (pilot phase) and 2007–2010 (main phase). People aged 40–69 years who lived within reasonable travelling distance (25 km) of one of the 22 assessment centers in England, Scotland, and Wales were identified from National Health Service patient registers and invited to attend an assessment center. Permission for access to patient records for recruitment was approved by the Patient Information Advisory Group (subsequently replaced by the National Information Governance Board for Health and Social Care) in England and Wales, and the Community Health Index Advisory Group in Scotland. At the UK Biobank assessment centers, a touchscreen questionnaire was used to collect information on sociodemographic characteristics, diet and other lifestyle exposures, general health, and medical history. Anthropometric measurements were obtained using standardized procedures.

This study was conducted according to the guidelines in the Declaration of Helsinki and all procedures involving human participants were approved by the North West Multi-center Research Ethics Committee. All participants gave informed consent to participate in the UK Biobank, and be followed up. The UK Biobank protocol is available online (https://www.ukbiobank.ac.uk/media/3sbeknnz/ukbiobank_protocol.pdf).

The UK Biobank dataset for the current analysis included 8715 participants assessed over a mean of 9.11 years (range 2.1–17.3 years). Participants were excluded if, at baseline, they were; aged <60 years, taking anti-depressant or anti-parkinsonian medications, had a diagnosis of depression or bipolar disorder, concussion, dementia (defined by data field 42018), cancer diagnosis, diabetes diagnosis, or hypertension (coded as International Classification of Diseases (ICD) 9 or 10; or blood pressure >140/90; or self-reported taking blood pressure medication), or had alcohol consumption >14 units/week (Supplemental Figure 1). See Supplemental Table 1 for specific excluded medications and ICD exclusionary codes. Included participants were required to have baseline data and at least one further assessment visit.

### Assessment of coffee and tea intake

At baseline, participants were asked to report their average coffee intake in the last year: ‘how many cups of coffee do you drink each day’, and their average tea intake in the last year: ‘how many cups of tea do you drink each day’. These responses were transformed into tertiles – ‘never’, ‘moderate’ (1–3 cups/day), and ‘high’ (≥4 cups/day). Participants were excluded if they stated consumption >10 cups of coffee (*n* = 60) and/or >15 cups of tea (*n* = 94) per day.

### Cognitive assessment

Cognitive measures were self-administered through a computerized touchscreen interface.^8^ The tests were designed specifically for the UK Biobank to allow administration without examiner supervision.

#### Fluid intelligence

This task assessed the ability to solve 13 verbal and numeric reasoning problems. Each problem had five possible response options. The dependent variable was the total number of correct answers given (range 0–13) within a two-minute period, with higher scores indicating better performance.

#### Reaction time

This task was delivered in the style of the card game, ‘snap’, and requested participants to respond with a button press when they detected the appearance of a matching pair of symbols. The dependent variable was the mean response time in milliseconds across 12 matching-pair trials.

#### Numeric memory

In this task, a string of numbers was presented on the screen, which subsequently disappeared. Participants were then asked to enter the number string from memory, in the reverse order. This increased by one until the participant made an error, or they reached the maximum of twelve digits. The dependent variable was the maximum string length recalled correctly (possible range 0–12), with higher scores indicating better performance.

#### Pairs matching errors

In this task, six card pairs of symbols were presented on the screen in a random pattern. Cards were then turned face down on the screen, and participants were asked to locate as many symbol pairs as possible in as few attempts as possible. The dependent variable was the number of errors made during pairs matching (possible range 0–146).

The fluid intelligence task was added to the participant assessment part-way through the baseline assessment phase. The numeric memory task was added and subsequently removed due to time constraints. Therefore, the sample sizes for different outcomes vary (Supplemental Figure 1).

### Apolipoprotein E genotyping

UK Biobank genotyping was conducted by Affymetrix using a bespoke BiLEVE Axium array or using the Affymetrix UK Biobank Axiom array. All genetic data were quality controlled centrally by UK Biobank resources. More information on the genotyping processes can be found online (https://www.ukbiobank.ac.uk). As the presence of the Apolipoprotein E (*APOE*) ε4 allele is strongly associated with increased risk of AD and cognitive decline,^9^ *APOE* carrier status was included in data analysis, defined as the presence (one or two copies) or absence (zero copies) of the ε4 allele.

### Demographic data

Demographic data collected at baseline included age, sex, educational qualification (held a college/university degree or not), ethnicity (white or non-white), Townsend deprivation score (measure of socioeconomic status; based on postcode of habitation and calculated from levels of employment, home and car ownership, and household overcrowding in a given area^10^), and body mass index (BMI; anthropometric measurements taken by staff). Current medications were self-reported to the research nurse.

### Statistical analysis

All statistical analyses were performed using R version 4.3.2 for Macintosh. A *p*-value of 0.05 determined a significant result.

Means, standard deviations and percentages for demographic data are provided (Table 1). Skewness and kurtosis of the dependent variables were tested; pairs matching errors values were square-root transformed and reaction time values were log-transformed because of skewed distribution.

**Table 1.** Demographics of the UK Biobank participants at baseline, aged 60 and over, with full dataset required for current analyses.

Characteristic	N = 8715
Sex, male, *n* (%)	3453 (40)
Age, mean (SD)	67.8 (5.7)
BMI, mean (SD)	26.0 (3.8)
*APOE* ε4 allele carriers, *n* (%)	2320 (27)
Daily coffee consumption, *n* (%)	
High (≥4 cups)	1539 (18)
Moderate (1–3 cups)	5030 (58)
Never	2146 (25)
Daily tea consumption, *n* (%)	
High (≥4 cups)	4093 (47)
Moderate (1–3 cups)	3289 (38)
Never	1333 (15)
Townsend deprivation index, mean (SD)	−2.23 (2.57)
Qualification, College/University, *n* (%)	3453 (40)
Ethnicity, *n* (%)	
White/Caucasian	8501 (98)
Mixed	26 (0.3)
Asian	85 (1.0)
Black	20 (0.2)
Unknown	83 (1.0)

*APOE*, Apolipoprotein E (gene); BMI, body mass index; SD, standard deviation.

A series of linear mixed model (LMM) analyses (using maximum likelihood estimation and an unstructured covariance matrix) were conducted to examine the relationship between independent variable (coffee intake, tea intake; analyzed independently) and change in cognitive function. Independent variable, age, *APOE* ε4 allele carrier status, sex, ethnicity, educational qualification, Townsend deprivation score, and BMI were entered as main effects, participant as a random factor, and independent variable*time as an interaction term. The respective cognitive outcome was entered as the dependent variable, with separate models run for each cognitive outcome. False discovery rate (FDR) correction was applied using a threshold of 0.10^11^ to the highest order interaction term within each model (i.e., not to baseline associations).

## Results

### Coffee intake and cognitive performance

**Figure 1.**
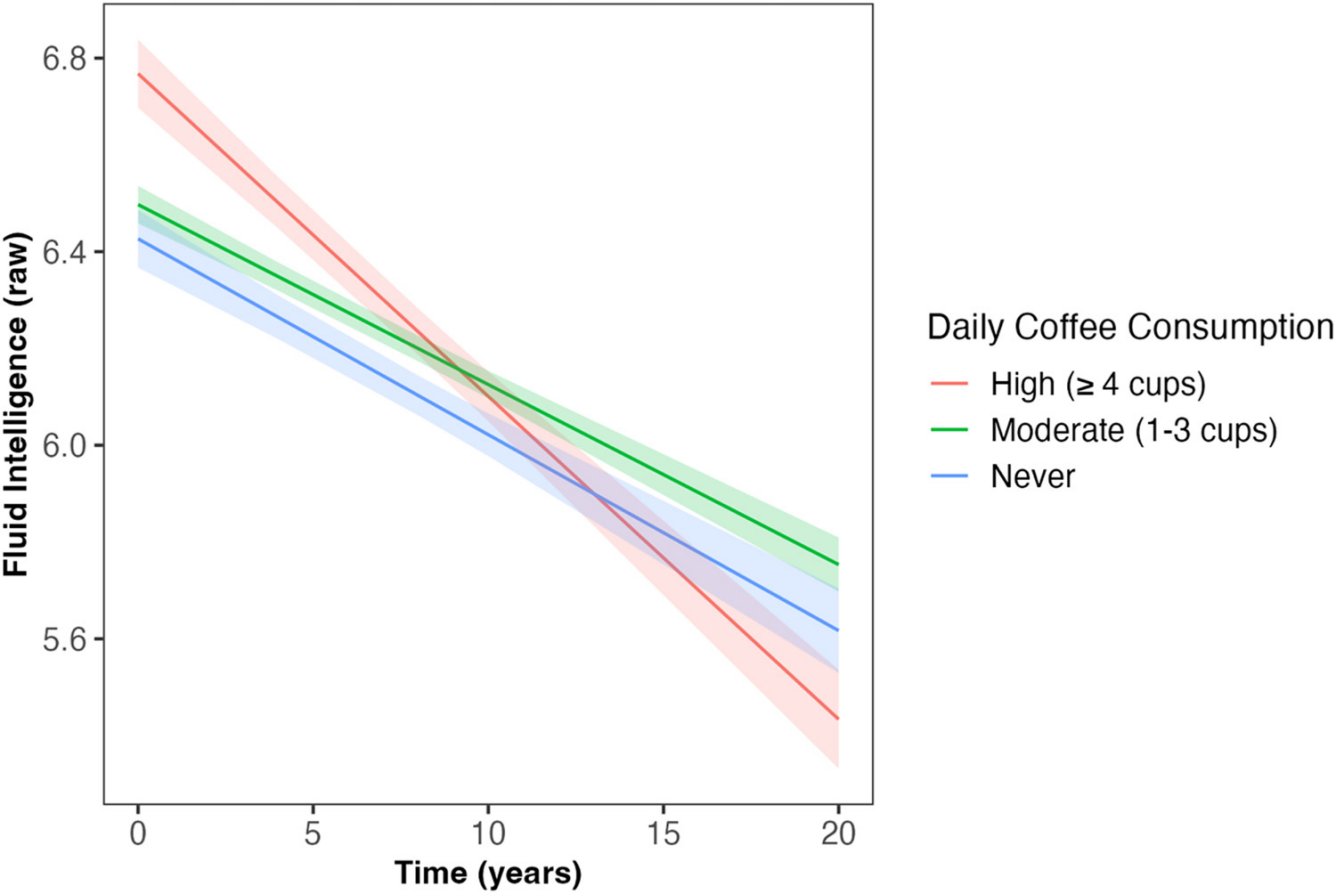
Daily coffee intake predicted the slope of decline in fluid intelligence across follow-up (β = −0.02, SE = 0.007, *p* = 0.001). Those ‘never’ consuming coffee and those with ‘moderate’ coffee consumption (1–3 cups/day) had slower decline in fluid intelligence compared to those with ‘high’ coffee consumption (≥4 cups/day; β = 0.06, SE = 0.02, *p* = 0.005; β = 0.07, SE = 0.02, *p* < 0.001, respectively). Shaded areas illustrate the standard error of the fitted values. Linear mixed model including covariates of age, sex, qualification, Townsend deprivation, ethnicity, *APOE* ε4 carriage status, and body mass index. *APOE*: Apolipoprotein E (gene); SE: standard error.

**Figure 2.**
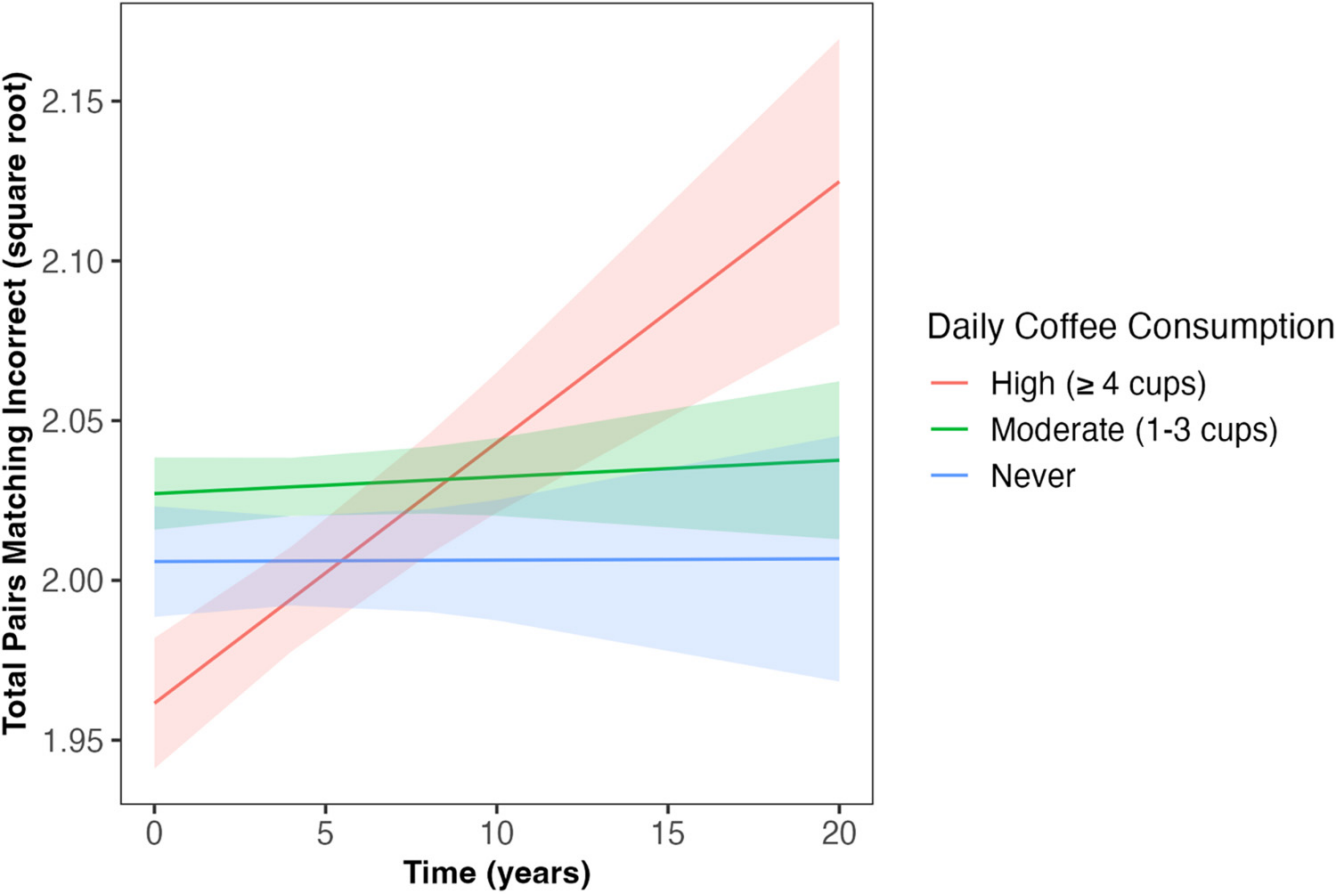
Daily coffee intake predicted change in number of pairs matching errors across follow-up (*β* = 0.01, *SE* = 0.007, *p* = 0.034). Those ‘never’ consuming coffee and those with ‘moderate’ coffee consumption (1–3 cups/day) had slower increases in the number of pairs matching errors compared to those with ‘high’ coffee consumption (≥4 cups/day; *β* = −0.05, *SE* = 0.02, *p* = 0.022; *β* = 0.05, *SE* = 0.02, *p* = 0.013, respectively). Shaded areas illustrate the standard error of the fitted values. Linear mixed model including covariates of age, sex, qualification, Townsend deprivation, ethnicity, *APOE* ε4 carriage status, and body mass index. *APOE*: Apolipoprotein E (gene); SE: standard error.

Daily coffee intake predicted the slope of decline in fluid intelligence and number of pairs matching errors across follow-up (*β* = −0.02, *SE* = 0.007, *p* = 0.001; *β* = 0.01, *SE* = 0.007, *p* = 0.034, respectively; Table 2); associations which survived correction for multiple comparisons. Those ‘never’ consuming coffee and those with ‘moderate’ coffee consumption (1–3 cups) had slower decline in fluid intelligence compared to those with ‘high’ coffee consumption (≥4 cups; *β* = 0.06, *SE* = 0.02, *p* = 0.005; *β* = 0.07, *SE* = 0.02, *p* < 0.001, respectively; Figure 1). Similarly, those ‘never’ consuming coffee and those with ‘moderate’ coffee consumption had slower increase in the number of pairs matching errors compared to those with ‘high’ coffee consumption (*β* = −0.05, *SE* = 0.02, *p* = 0.022; *β* = 0.05, *SE* = 0.02, *p* = 0.013, respectively; Figure 2). Coffee consumption was not associated with reaction time or numeric memory at baseline or across follow-up (Table 2).

**Table 2.** Linear mixed models of associations between coffee and tea intake and cognitive outcomes. Standardized β (standard error) shown.

	Fluid intelligence (n = 8061)	Reaction time (log) (n = 8704)	Pairs matching errors (sqrt) (n = 8715)	Numeric memory (n = 4170)
Coffee intake				
Coffee	**0.003** (0.010)**	−0.007 (0.008)	**−0.015** (0.008)**	−0.030 (0.015)
Coffee×Time	**−0.02** (0.007)***†	0.006 (0.005)	**0.014** (0.007)*†	−0.001 (0.013)
Tea intake				
Tea	**−0.029** (0.010)***	0.013 (0.008)	0.016 (0.008)	−0.030 (0.015)
Tea×Time	**0.017** (0.007)*†	−0.005 (0.005)	0.002 (0.006)	0.008 (0.013)

Covariates for all models include age, sex, qualification, Townsend deprivation, ethnicity, *APOE* ε4 carriage status, and body mass index. Abbreviations: *APOE*, Apolipoprotein E (gene); FDR, false discovery rate; sqrt, square-root transformation.

* *p* < 0.05, ** *p* < 0.01, *** *p* < 0.001, uncorrected.

†Significant after correction for multiple comparisons; the FDR correction with a threshold of 0.10 was applied to the interaction term within each predictor variable.

### Tea intake and cognitive performance

**Figure 3.**
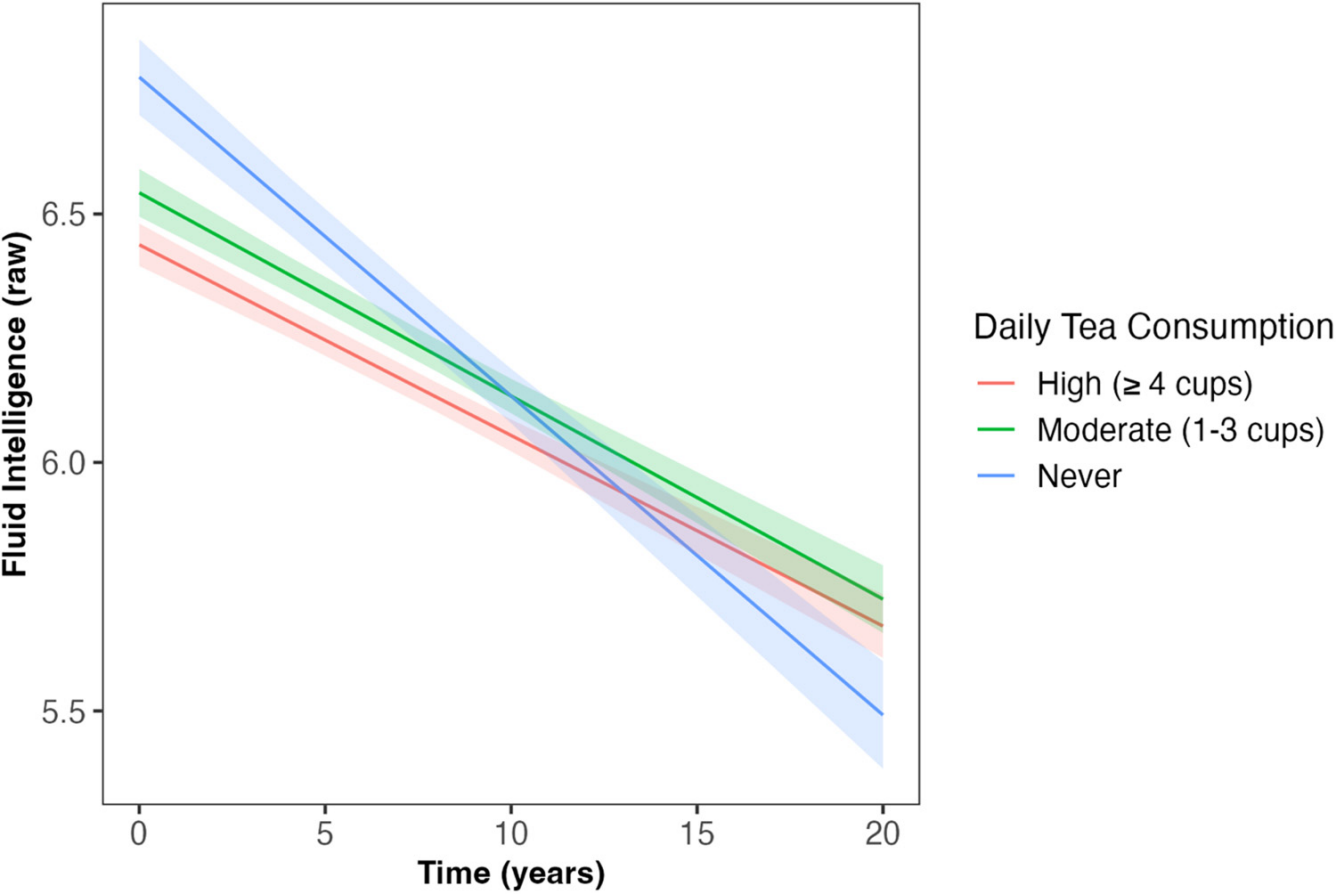
Daily tea intake predicted the slope of decline in fluid intelligence across follow-up (β = 0.017, SE = 0.007, p = 0.015). Those who had ‘moderate’ (1–3 cups/day) and ‘high’ (≥4 cups/day) tea consumption had less decline in fluid intelligence compared to those who ‘never’ drank tea (*β* = 0.06, *SE* = 0.02, *p* = 0.009; *β* = 0.06, *SE* = 0.02, *p* = 0.004, respectively). Shaded areas illustrate the standard error of the fitted values. Linear mixed model including covariates of age, sex, qualification, Townsend deprivation, ethnicity, *APOE* ε4 carriage status, and body mass index. *APOE*: Apolipoprotein E (gene); SE: standard error.

Daily tea intake predicted the slope of decline in fluid intelligence across follow-up (*β* = 0.017, *SE* = 0.007, *p* = 0.015, Table 2, Figure 3), such that those who had ‘moderate’ and ‘high’ tea consumption showed less decline in fluid intelligence compared to those who ‘never’ drank tea (*β* = 0.06, *SE* = 0.02, *p* = 0.009; *β* = 0.06, *SE* = 0.02, *p* = 0.004, respectively); associations which survived correction for multiple comparisons. However, at baseline, those with ‘moderate’ and ‘high’ tea consumption showed worse fluid intelligence performance than those who ‘never’ drank tea (*β* = −0.03, *SE* = 0.03, *p* = 0.011; *β* = −0.08, *SE* = 0.03, *p* < 0.001, respectively). Tea consumption was not associated with reaction time, number of pairs matching errors, or numeric memory at baseline or across follow-up (Table 2).

## Discussion

The aim of this study was to investigate the relationship between habitual coffee and tea intake and rates of decline in a range of cognitive outcomes, over a mean of 9.11 years, in 8715 individuals free of dementia at baseline. Our results showed ‘high’ coffee consumption and ‘never’ drinking tea were associated with poorer cognitive outcomes. Those ‘never’ consuming coffee and those with ‘moderate’ coffee consumption (1–3 cups/day) demonstrated slower decline in fluid intelligence and slower increase in the number of pairs matching errors compared to those with high coffee consumption (≥4 cups/day). Additionally, those who had ‘moderate’ and ‘high’ tea consumption showed less decline in fluid intelligence compared to those who ‘never’ drank tea. Whilst the association with the number of pairs matching errors was only observed for coffee intake, and not tea intake, the data could be noisy (as implied from the wide confidence intervals) and the effect of tea consumption on the number of pairs matching errors potentially isn’t as robust and, therefore, is not showing statistical significance.

Similar to the longitudinal findings of the current study, higher baseline coffee consumption has been shown to be associated with slower decline in executive function, attention, and a Preclinical Alzheimer's Cognitive Composite in a cohort of cognitively unimpaired older adults from the Australian Imaging, Biomarkers and Lifestyle study.^12^ However, the majority of extant literature examines dementia incidence or global cognitive decline as opposed to decline in distinct cognitive outcomes. For example, van Gelder et al.,^13^ conducted a 10-year prospective cohort study in males (*n* = 676) using the Mini-Mental State Examination (MMSE; a measure of global cognition) and observed an inverse and J-shaped association between the number of cups of coffee consumed and cognitive decline, with the least cognitive decline for three cups/day of coffee (0.6 points). In contrast to our findings, a Finnish study of 2606 middle-aged twins assessed in 1975 and 1981, with a median follow-up of 28 years, found that coffee consumption was not an independent predictor of cognitive performance in old age.^14^ Similarly, a study of Japanese males (*n* = 3494) born between 1900 and 1919 and living in Hawaii in 1964 found no association between coffee intake and the risk of cognitive impairment 25 years later. Coffee intake was measured using a 24-h dietary recall, and the specific inclusion criteria of the cohort limit the generalizability of these results.^15^ In both studies, cognition was not assessed in specific domains but through screening tools, the Cognitive Abilities Screening Instrument (CASI), a screen for potential dementia cases (TELE), and the Telephone Interview for Cognitive Status (TICS).

Tea consumption has been more consistently associated with better cognitive performance or reduced decline^16,17^ compared with coffee consumption. A Chinese study of 4657 individuals (≥55 years) who underwent at least two cognitive assessments over nine years, showed tea consumption was associated with reduced global cognitive decline, and ≥4 cups/day was inversely associated with self-reported poor memory and memory decline. The cognitive assessment used in the aforementioned study only comprised a global cognitive assessment, the TICS-modified, and one question each for assessing self-reported poor memory and memory decline.^18^ A further Chinese study in a different cohort, provided additional evidence that daily tea consumption (green tea and non-green tea) has a protective effect on cognitive function compared to no tea or occasional tea consumption.^19^ Participants (*n* = 76,270) were cognitively unimpaired, aged between 65 and 105 years at baseline, and completed up to six cognitive assessments over 16 years. Again, the cognitive assessment only comprised a global assessment, the MMSE.

It is still unknown which specific components of coffee and tea are responsible for the positive outcomes observed in this study and others, however given both beverages showed positive responses, it could be hypothesized that it is the same constituent in both; one such constituent is caffeine. Given our results observed an upper limit of beneficial coffee consumption of 3 cups/day, with no upper limit of beneficial tea consumption per day, caffeine furthermore seems a likely option with tea's comparatively lower caffeine content compared to coffee (approximately 45 mg/cup black tea, 28 mg/cup green tea, and 95 mg/cup coffee)^20^ and thus our sample being less likely to meet the equivalent caffeine amount in tea per day, which would be approximately 6–10 cups/day. *In vivo* and *in vitro* studies show evidence that caffeine has long-term, neuroprotective effects against AD through multiple mechanisms including the altering of amyloid precursor protein processing to a non-amyloid pathway, reducing AD burden and cognitive decline.^21,22^ Furthermore, caffeine's neuroprotective effect could be due to its ability as a non-selective adenosine receptor antagonist, reducing inflammatory processes associated with AD.^23,24^ Blocking the binding of adenosine to its receptor by caffeine can also produce acute effects on cognition, as this affects the release of neurotransmitters such as norepinephrine, dopamine, acetylcholine, serotonin, glutamate, and gamma-aminobutyric acid. An influx in these neurotransmitters alters mood, memory, alertness, and cognitive function.^25^ However, in the context of the current study, these ideas of protective constituents are speculative given this cannot be assessed using our research paradigm.

There are several limitations to consider when interpreting our findings. Although we adjusted for known potential confounding factors there is a possibility of residual confounding due to other factors which were not measured. For example, high coffee intake could be associated with stress due to work or nightshifts, or sleep disruption. Measurement error or recall bias may affect the dietary data, though coffee and tea intake is less likely to be misreported due to its habitual nature over time. Additionally, participants with dementia at baseline were excluded to minimize the risk of measurement error or recall bias. Data on coffee or tea consumption during mid-life were not collected, so the potential positive or negative effects of coffee and tea intake at that stage cannot be assessed in this study. It was also not possible to determine potential consequences of varying methods of coffee preparation (e.g., decaffeinated coffee, brewing method, with or without milk or sugar etc.) on the associations observed. Of particular note, protein in milk has been shown to interact with phenolic compounds contained in coffee and to negatively influence coffee's antioxidant properties.^26^ Furthermore, tea intake was not quantified individually for types of tea, such as black, white, or green.

Another limitation concerns the cognitive tests which were non-standard, bespoke tests. The reaction time and memory tests were brief, including smaller numbers of trials than is typical for reaction time tests.^27^ The memory test was based on the recall of a single 12-item matrix with six pairs of stimuli. This is both a brief and unusual type of test in the field of declarative memory; declarative memory tests such as word lists and paragraph recall are more widely used.^28^ The fluid intelligence test had only 13 items, some of which had floor/ceiling effects, but it had acceptable internal consistency.^29^ With more in-depth cognitive testing we might expect to find additional coffee and tea intakes linked to changes in cognitive performance over time. However, despite the nature of the UK Biobank cognitive tests, they have been shown to correlate moderately-to-strongly with well-validated cognitive tests that were designed to assess the same cognitive domain.^30^ Strengths of our study include the long duration of follow-up, large number of participants, and comprehensive characterization of the cohort, including concurrent assessment of multiple outcomes of cognition.

Our findings reinforce the concept that ‘moderate’ habitual coffee intake and ‘moderate’ and ‘high’ habitual tea intake may be a protective factor against cognitive decline with increasing age. There is a need for randomized controlled trials to establish causal relationships leading to evidence-based recommendations about the risks and benefits of coffee and tea intake for cognitive function in aging populations.

## Supplemental Material

sj-docx-1-alz-10.1177_13872877251361058 - Supplemental material for Moderate coffee and tea consumption is associated with slower cognitive decline Supplemental material, sj-docx-1-alz-10.1177_13872877251361058 for Moderate coffee and tea consumption is associated with slower cognitive decline by Stephanie R Rainey-Smith, Kelsey R Sewell, Belinda M Brown, Hamid R Sohrabi, Ralph N Martins, Samantha L Gardener in Journal of Alzheimer's Disease
