# Supplementary material for: Moderate coffee and tea consumption is associated with slower cognitive decline
Source: J Alzheimers Dis. 2025 Jul 21;107(1):170–8. doi: 10.1177/13872877251361058 (PMC12361686; doi:10.1177/13872877251361058)
Supplement: sj-docx-1-alz-10.1177_13872877251361058 - Supplemental material for Moderate coffee and tea consumption is associated with slower cognitive decline [file sj-docx-1-alz-10.1177_13872877251361058.docx]

**Supplemental Material**

**Moderate coffee and tea consumption is associated with slower cognitive decline**

**Supplemental Table 1.** Study exclusion criteria.

| **Exclusion criteria** | **Description** | **Relevant Field ID(s)** | **ICD9 and ICD10 codes (if relevant)** | **Medication codes (if relevant)** |
| --- | --- | --- | --- | --- |
| Aged <60 baseline |  | 34 |  |  |
| All cause dementia baseline | Algorithmically defined outcome | 42018 |  |  |
| Concussion baseline |  | 41270  41271 | S060  S0600  S0601  850  8509 |  |
| Diagnosed bipolar or depression | Included individuals with either NA or “No bipolar or depression” | 20126 |  |  |
| Anti-depressant medications |  | 20003 |  | Citalopram: 1140921600  Escitalopram: 1141180212  Fluoxetine: 1140879540  Paroxetine: 1140867888  Sertraline: 1140867878  Duloxetine 1141200564  Fluvoxamine 1140879544  Amitriptyline 1140879616  Venlafaxine 1140916282, 1140916288  Prozac 1140867876 (fluoxetine) |
| Anti-parkinsonian medications |  | 20003 |  | Sinemet: 1140872420, 1140872434, 1141164872  Amantadine: 1140879644  Bromocriptine: 1140868866  Pergolide: 1140879660  Selegiline: 1140879668 |
| Cancer diagnosis | Cancer diagnosed by a doctor | 2453 |  |  |
| Hypertension baseline | Defined as any of the following:  Reported on ICD9 or 10; systolic blood pressure ≥140 or diastolic blood pressure ≥90;  Self-reported taking blood pressure medication | 41270  41271  93  94  6177  6153 | I10, I11, I12, I13, I15, O10, 401, 402, 403, 404, 405 |  |
| Diabetes baseline | Diabetes diagnosed by a doctor | 2443 |  |  |
| Excessive alcohol consumption | >14 standard units/week | 1568  1578  1588  1598  1608 |  |  |

ICD: International Classification of Diseases; ID: identification; NA: not available

**Supplemental Figure 1.** Flow diagram indicating number of participants with data available for inclusion for each analysis of coffee/tea intake and cognitive performance (Pairs matching; Fluid intelligence; Reaction time; Numeric memory).


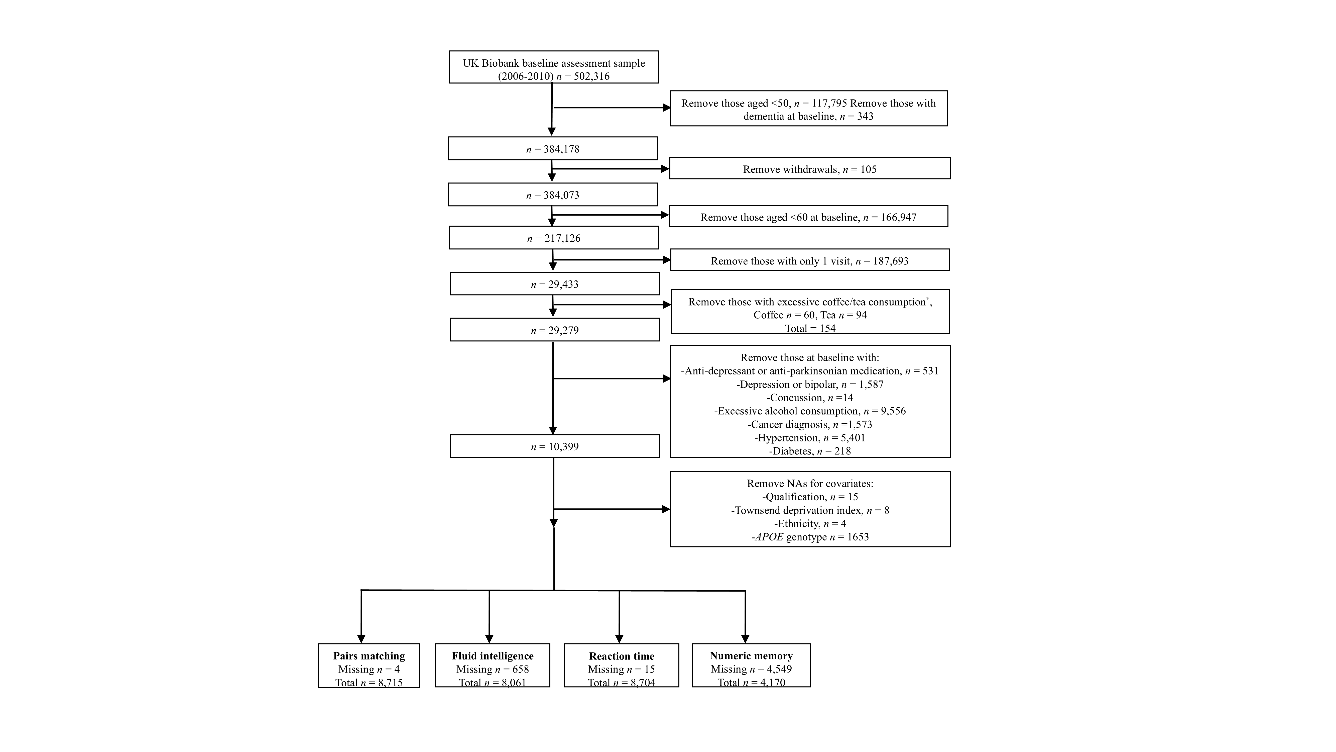
 *APOE*: Apolipoprotein E (gene); NA: not available.

^*^Excessive coffee/tea consumption defined as >10 cups of coffee and/or >15 cups of tea per day.
